# Lake Ontario salmon (*Salmo salar*) were not migratory: A long-standing historical debate solved through stable isotope analysis

**DOI:** 10.1038/srep36249

**Published:** 2016-11-08

**Authors:** Eric J. Guiry, Suzanne Needs-Howarth, Kevin D. Friedland, Alicia L. Hawkins, Paul Szpak, Rebecca Macdonald, Michelle Courtemanche, Erling Holm, Michael P. Richards

**Affiliations:** 1 Department of Anthropology, University of British Columbia, 6303 NW Marine Dr., Vancouver, BC V6T1Z1, Canada; 2 Perca Zooarchaeological Research & Trent University Archaeological Research Centre, 1600 West Bank Dr., Peterborough, ON K9L 0G2, Canada; 3 National Marine Fisheries Service, 28 Tarzwell Dr., Narragansett, RI, 02882, USA; 4 Archaeology program, School of the Environment, Laurentian University, 935 Ramsey Lake Rd., Sudbury, ON P3E 2C6, Canada; 5 Department of Anthropology, Trent University, 1600 West Bank Dr., Peterborough, ON K9L 0G2, Canada; 6 Ostéothèque de Montréal, Université de Montréal, 2900 Blvd. Edouard-Montpetit, Montréal, QC H3T 1J4, Canada; 7 Department of Natural History, Royal Ontario Museum, 100 Queen’s Park, Toronto, ON M5S 2C6, Canada; 8 Department of Archaeology, Simon Fraser University, 8888 University Dr., Burnaby, BC V5A1S6, Canada

## Abstract

Lake Ontario once supported a large complex of Atlantic Salmon (*Salmo salar*) populations that became extinct prior to scientific study. Since the 1860s, research efforts to conserve and reintroduce a sustainable population of Atlantic Salmon have focused on determining whether Lake Ontario’s original salmon populations had migrated to the Atlantic Ocean as part of their lifecycle (anadromy), stayed in the lake year-round (potamodromy), or both. We used stable carbon, nitrogen, and sulfur isotope analyses of archaeological bones and historical museum-archived salmon scales to show that the original salmon populations from Lake Ontario completed their entire lifecycle without migrating to the Atlantic Ocean. With a time depth of more than 500 years, our findings provide a unique baseline with significant potential for informing modern restocking and conservation efforts.

Before 1850, Lake Ontario, the most easterly of the North American Great Lakes, supported a unique complex of Atlantic Salmon populations (*Salmo salar*; hereafter Lake Ontario salmon) that formed the basis of an immense subsistence and commercial freshwater fishery^1^,^2^. Salmon had been exploited in the Lake Ontario and St. Lawrence River watersheds since at least the early Holocene^3^,^4^,^5^; however, by 1900, they had disappeared^1^,^2^. In addition to overharvesting, historical observers attributed the decline of Lake Ontario salmon to other human impacts, such as escalating river pollution, poaching, deforestation, and a loss of spawning habitat^6^. The precipitous and highly visible decline of the Lake Ontario salmon gained broad significance as a catalyst for the scientific development of North American fisheries management^6^,^7^.

When a vital aquatic resource is lost, baseline biological information about the behaviours of the original population can be a crucial asset not only for historical ecologists but also for conservation biologists and their reintroduction efforts^8^,^9^. Following the idea that it was behavioural, rather than biological^2^,^10^ differences that made Lake Ontario salmon unique among Atlantic Salmon^11^,^12^, generations of researchers have sought out and debated clues about their behavioural ecology in relation to other populations of Atlantic Salmon^1^,^2^,^11^,^12^,^13^,^14^,^15^,^16^,^17^,^18^,^19^,^20^,^21^,^22^,^23^. Over the past 150 years, analyses of historical observations and opinions have provided a basis for multiple, and often contradictory, interpretations of Lake Ontario salmon behavioural ecology (for a review, see Supplementary Information). The most controversial question^1^,^2^,^13^, which remained unanswered^11^, has been whether Lake Ontario salmon migrated to the Atlantic Ocean as part of their lifecycle (anadromy), stayed in the lake year-round (potamodromy), or both.

We use stable carbon (*δ*^13^C), nitrogen (*δ*^15^N), and sulphur (*δ*^34^S) isotope analyses of archaeological bone and historical scale remains from the extinct populations of Lake Ontario salmon to reveal key aspects of their behavioural ecology. Isotopic analyses of salmon remains from Iroquoian and European sites (Fig. 1), spanning the period 1300 to 1840 AD along the northwest shore of Lake Ontario and the upper St. Lawrence River, provide direct evidence of salmon migratory behaviour and reveal that their behavioural ecology was more complex than historical eyewitness accounts describe^1^,^2^,^14^,^15^,^16^,^20^,^23^. Our data provide a new baseline that may be helpful to salmon reintroduction and conservation efforts in the region^24^,^25^,^26^,^27^,^28^. We expected that the migratory behaviour of Atlantic Salmon in Lake Ontario could be revealed through analyses of their isotopic values, which can indicate if they had lived primarily in a freshwater (low *δ*^13^C and *δ*^34^S values) or marine (high *δ*^13^C and *δ*^34^S values) environment. Our hypothesis was that Lake Ontario salmon would follow either an anadromous or a potomodromous behavioural strategy.

## Results

Stable isotope results from archaeological and historical salmon (*n* = 74 for *δ*^13^C and *δ*^15^N; *n *= 25 for *δ*^34^S) materials are presented in Fig. 2 (for data, see Tables S7 and S8 in Supplementary Information). Results from analyses of scale circuli spacing for five museum-archived salmon skin mounts (Fig. 3) support museum documentation of specimen origin indicating that three fish came from Lake Ontario salmon and two came from St. Lawrence River Atlantic Salmon (Supplementary Information).

Historical scales produced *δ*^13^C values consistent with modern isotopic baseline datasets for freshwater smolt and marine adult salmon scales, respectively^29^,^30^,^31^. Wide separation between *δ*^13^C and also *δ*^34^S values of fish with different migratory behaviours indicates that: 1) salmon with *δ*^13^C and *δ*^34^S values below −19‰ and +12‰, respectively, completed their entire lifecycle as freshwater residents in Lake Ontario, and 2) salmon with a *δ*^13^C values above −17‰ and *δ*^34^S values above +14‰ made a round trip from their natal stream down the St. Lawrence River to the Atlantic Ocean and back over the course of their lives. These results are consistent with other studies that have observed a similar bimodal distribution in *δ*^13^C and *δ*^34^S between anadromous and potamodromous fish^32^,^33^,^34^,^35^. Analyses from other modern taxa, including similar pelagic consumers, distributed across Lake Ontario also have low *δ*^13^C and *δ*^34^S values, suggesting that regional variability should not influence isotopic signatures for Lake Ontario resident salmon to the extent that the anadromous/potamodromous distinction would be obscured^36^,^37^,^38^,^39^.

Archaeological salmon bone *δ*^13^C and *δ*^34^S values fit well within the isotopic ranges (as defined by *δ*^13^C and *δ*^34^S value ranges of the modern and/or historical salmon scale isotope baselines) expected for either potamodromous or anadromous fish. Given the great abundance of salmon in the tributaries of Lake Ontario evident in the historical record prior to the 1850s^2^, it is unlikely that salmon bones from sites near western Lake Ontario would originate from salmon traded from another region further afield and, therefore, it is highly likely that these samples represent individuals from the Lake Ontario salmon populations. A pattern emerges when the regional archaeological context of salmon bones is compared with *δ*^13^C and *δ*^34^S values. Whereas all salmon from sites near the western side of Lake Ontario produced a clear potamodromous signal (*n *= 50, average *δ*^13^C = −20.0 ± 0.4‰; *n *= 17, average *δ*^34^S = +8.7 ± 1.6‰), salmon from sites on the upper St. Lawrence (between Montréal and Lake Ontario) produced evidence for a mix of both potamodromous (*n *= 7, average *δ*^13^C = −22.2 ± 0.3‰; *n *= 2, average *δ*^34^S = +3.6 ± 0.1‰) and anadromous (*n *= 8, average *δ*^13^C = −15.7 ± 0.6‰; *n *= 5, average *δ*^34^S = +15.1 ± 1.0‰) strategies (Fig. 2). It is possible, albeit less parsimonious, that some anadromous individuals from the upper St. Lawrence could have reached this site through upriver trade rather than migration. It is also noteworthy that some of the samples (*n *= 7) from the St. Lawrence River area have stable isotope values indicating potamodromous behaviour. In comparison with the dataset from salmon from sites near the west side of Lake Ontario, these individuals produce much lower *δ*^13^C and *δ*^34^S values, suggesting that they may have come from a different but as yet unknown potamodromous Atlantic Salmon population, possibly from a local freshwater lake.

Historical salmon scale and archaeological bone collagen samples produced a wide range (5.0‰) of *δ*^15^N values that appear to cluster in two groups (Fig. 2). Whereas most of the archaeological bone samples with a potamodromous isotope signal (*n *= 46 of 50) showed a relatively tight clustering, with *δ*^15^N values that averaged +10.3 ± 0.3‰ (range = 1.2‰), both historical Lake Ontario salmon scales and archaeological bones from the Steven Patrick site had significantly elevated *δ*^15^N values, which averaged +13.1 ± 0.6‰ (*n *= 9, range = 1.1‰). Although further analyses will be necessary to explain these differences in values, we hypothesize that these differences reflect temporal or geographical variation in salmon trophic level; differences in *δ*^15^N values at the base of the food web in Lake Ontario; or, possibly, trade in fish from another landlocked salmon population. The latter possibility, however, would be unlikely given the local abundance of salmon from Lake Ontario and because no other landlocked salmon population was historically known in the region. Scale and bone collagen samples with an anadromous *δ*^13^C and *δ*^34^S signal (*n *= 10) also showed a wide range of variation in *δ*^15^N values (3.9‰), but this was more evenly distributed (average *δ*^15^N = +10.9 ± 1.2‰) and is in line with modern scale baseline data from anadromous salmon.

## Discussion and Conclusion

The archaeological results follow the isotopic pattern expected for anadromous and potamodromous behaviours and provide the first direct evidence for assessing the longstanding debate over the migratory behaviour of Lake Ontario’s extinct Atlantic Salmon populations. Remarkably uniform stable carbon and sulphur isotope data for salmon bones from nineteenth-century Euro-Canadian and pre-contact Aboriginal archaeological sites around western Lake Ontario confirm that this unique salmon stock behaved potamodromously and also show that baseline isotopic values for top pelagic predators remained stable (particularly for *δ*^13^C) for at least the last 500 years prior to European settlement. This evidence supports historical hypotheses (see Supplementary Information) suggesting that, although Lake Ontario salmon may have encountered no physical barrier to returning to the Atlantic Ocean, Lake Ontario was sufficiently large and productive that unique local salmon populations evolved a behavioural adaptation to complete their entire life cycle in freshwater, without undertaking the metabolically costly journey up the St. Lawrence River. Moreover, the unanimous agreement of all Lake Ontario salmon bone *δ*^13^C and *δ*^34^S data from sites spread over roughly 100 km and spanning more than 500 years suggests that potamodromy was not only the dominant but also a stable behavioural strategy from at least the beginning of the Little Ice Age until the population’s extinction. This suggests that the salmon populations spawning in the tributaries entering north-western shore of Lake Ontario (i.e., the end farthest from the St. Lawrence River) were relatively isolated with respect to genetic admixture from their anadromous counterparts.

Our dataset also reveals clear evidence, at least with respect to geographical proximity, for the potential mixing of Lake Ontario resident salmon and anadromous salmon travelling up the St. Lawrence River. Limited *δ*^13^C and *δ*^34^S data available from sites on the upper St. Lawrence River provides a surprisingly clear example of fish with anadromous isotopic signatures that are, in the context of the length of the entire St. Lawrence watercourse, only a short distance from Lake Ontario. It is plausible that, having travelled most of the length of the St. Lawrence River, these salmon could have completed the journey, perhaps to make use of Lake Ontario’s eastern tributaries on the New York state side of Lake Ontario for spawning. Regardless, these data highlight the potential for future analyses focusing on salmon from sites around eastern Lake Ontario to explore salmon population mixing, which could have important implications, both genetically and behaviourally, for understanding and reviving or replacing Lake Ontario’s unique salmon.

Returning to the issue of conservation, our results provide important contextual information for ongoing and future attempts to reintroduce a sustainable population of Atlantic Salmon to Lake Ontario. One strategy that has been proposed to repopulate Lake Ontario with Atlantic Salmon is to use a source population with a similar range of behavioural traits, in particular, similar migratory behaviour. Up until now there has always been some uncertainty around the migratory strategy of the Lake Ontario populations. Our research shows unequivocally that these fish were potamodromous, rather than anadromous.

## Materials and Methods

### Methodological Approach

Established biogeochemical methods that have been used to identify marine and freshwater migratory behaviours in modern fish populations, such as strontium isotope or calcium/strontium ratio analyses^40^, could be problematic for archaeological contexts where concentrations of these elements may be prone to diagenetic alteration in bone mineral^41^, particularly for more-porous fish bone. In contrast, *δ*^13^C, *δ*^15^N, and *δ*^34^S analyses of fish bone collagen have well-established criteria for assessing sample integrity in archaeological contexts where diagenesis may be a problem^42^,^43^,^44^,^45^ and are also well suited for reconstructing ecosystem nutrient relationships^46^,^47^,^48^ as well as identifying marine and freshwater migratory behaviour^32^,^33^,^34^,^35^.

### Sample Description

**Figure 1:**
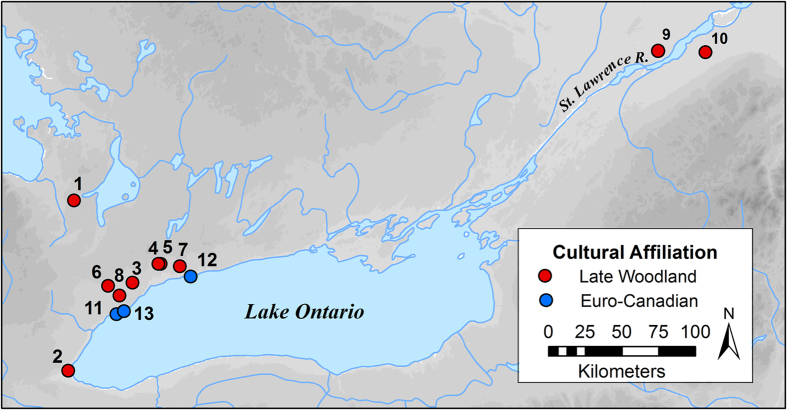
Map showing locations of archaeological sites. 1 – Steven Patrick, 2 – Skyway, 3 –Robb, 4 – Joseph Picard, 5 – Yatsihsta’, 6 – Bathurst St., 7 – Grandview, 8 – Moatfield, 9 – Summerstown Station, 10 – Mailhot-Curran, 11 – Bishop’s Block, 12 – Trull, 13 – Ashbridge. For cultural affiliation, Late Woodland includes Iroquoian sites dating from approx. AD 1300–1550, and Euro-Canadian includes historical European settlement sites dating from approx. AD 1790–1900. Data from Grandview and Moatfield are from published literature^53^. Figure created by AH using ArcGIS Desktop, Release 10 (www.esri.com).

**Figure 2:**
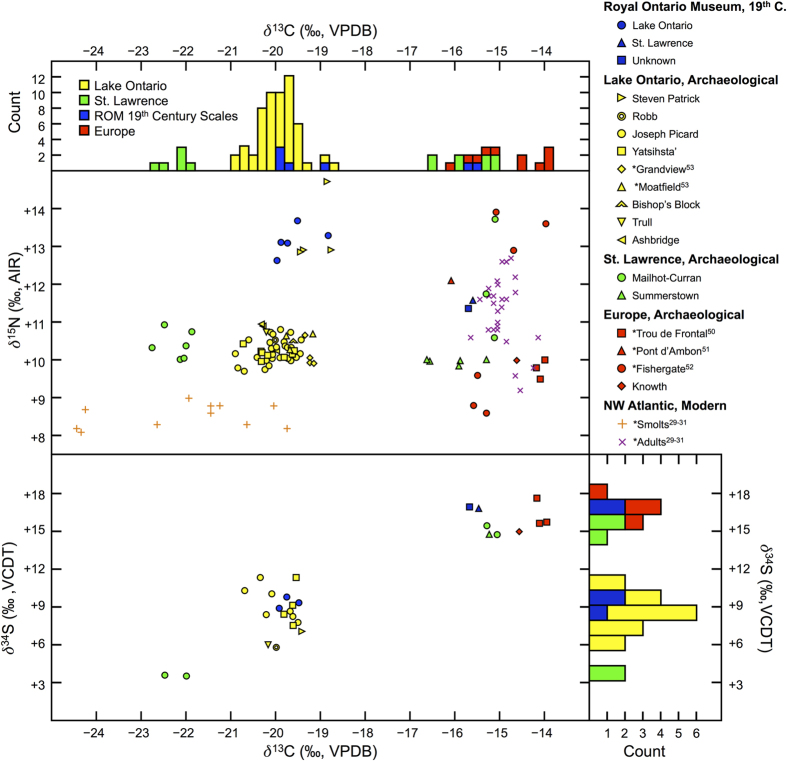
Bivariate plots of *δ*^13^C vs. *δ*^15^N (top) and *δ*^13^C vs. *δ*^34^S (bottom) values and histograms showing separation of *δ*^13^C (left) and *δ*^34^S (right) values for salmon from different regions. Comparative salmon data from modern scales^29^,^30^,^31^ as well as European^50^,^51^,^52^ and North American^53^ archaeological bone are denoted by an asterisk. Modern scale *δ*^13^C data has been adjusted by +1.25‰ to account for historical changes in environmental *δ*^13^C^59^,^60^. Data points for modern smolt (*n* = 442) and adult (*n *= 1866) salmon scales are average values for separate sample populations (including between 21 and 249 individual fish) in Europe, Greenland, and North America.

**Figure 3:**
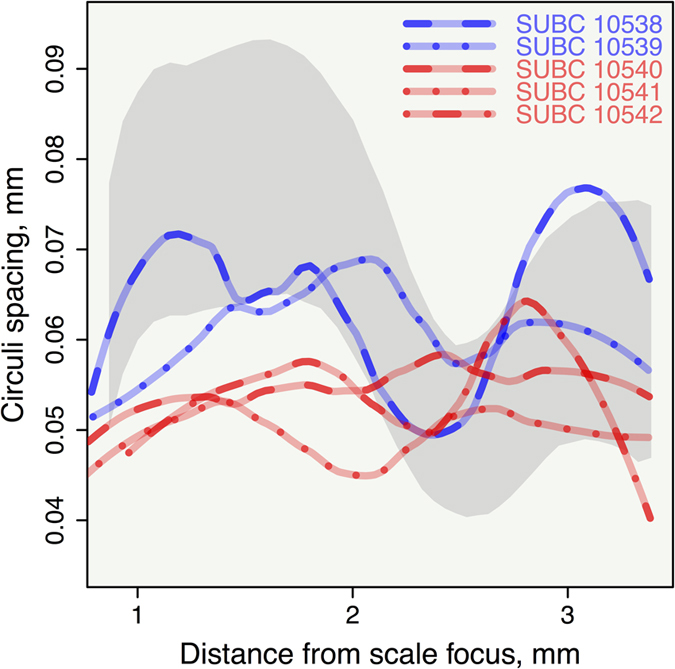
Circuli spacing versus distance from the scale focus for a composite representation of sea-run Miramichi Atlantic salmon (grey band is mean ± SD for all observations)^55^ and the loess smoothing line plots of circuli spacing measurements for scales from nineteenth-century museum-archived salmon skin mounts from St. Lawrence River (SUBC 10538 and 10539) and Lake Ontario (SUBC 10540, 10541, and 10542) specimens. Samples are coded by colour.

Lake Ontario salmon samples came from: 1) archaeological bones from 9 sites near western Lake Ontario and 2 sites near the upper St. Lawrence River, all from contexts dating between 1300 and 1840 AD (Fig. 1; Table S1 in Supplementary Information) as well as 2) historical scales from 7 nineteenth-century Atlantic Salmon skin mounts archived at the Royal Ontario Museum (Table S2 in Supplementary Information). Taxonomic identifications for salmon bones were made by zooarchaeologists as part of academic research or Cultural Resource Management archaeological projects. Taxonomic identifications were reconfirmed based on visual and morphological comparisons with a modern reference collection by three ichthyoarchaeological experts (SN, AH, and MC) for this research. Where possible, bone samples were selected based on Minimum Number of Individual counts per archaeologically unique context to ensure that each sample represents a distinct individual salmon. In the few instances where this was not possible, samples were taken from separate excavation units to minimize the likelihood of sampling the same individual salmon multiple times. Our sampling efforts identified a total 74 confirmed archaeological *S. salar* bones from relevant archaeological contexts that were made available for isotopic analyses. Because the organic components of both bone and scales are composed primarily of Type I collagen, these two sample types are directly comparable, and both represent long-term dietary intake^49^. Comparative data from modern Atlantic Salmon scales^29^,^30^ as well as European^50^,^51^,^52^ and North American^53^ archaeological bones were sourced from previously published studies and are supplemented by new analyses of a single European Atlantic Salmon individual from an Early Christian context from the site of Knowth in Ireland. Morphological analyses of scales from 5 of the 7 nineteenth-century Atlantic Salmon skin mounts were used to provide a second line of evidence for salmon origin and were conducted at the National Marine Fisheries Service (Narragansett, RI, USA) using established methods^54^,^55^ (Supplementary Information).

### Sample Preparation

Scales were cleaned prior to isotopic analyses with a scalpel and sonicated in deionized water for 15 min, in acetone for 5–10 min, and again in deionized water for 3 × 15 min to remove adhering fats, tissue, guanine, and other potential contaminants. Cleaned scales were soaked in 1.2 M HCl for 2 min^56^ followed by additional rinses in an ultrasonic bath of deionized water 2 × 3–5 min. Demineralizing of the external plate should loosen it from the underlying collagen-rich fibrillar plate, thus helping to ensure the complete removal of any contaminants that may have been applied to or settled upon the external surfaces of the salmon skin mounts.

Bones were cleaned of surface materials and cut into small chunks (c. 3 mm^3^). Samples were then treated three times with 2:1 chloroform-methanol in an ultrasonic bath (5–10 min each) to remove residual lipids^42^,^57^. Sample demineralization was then achieved by soaking samples in 0.5 M HCl. Samples were then rinsed in Type I water to neutrality, and base-soluble contaminants were removed by treating samples with 0.1 M NaOH several times in an ultrasonic bath (solution refreshed every 15 min until solution remained clear). Samples were again rinsed in Type I water to neutrality and then solubilized in 10^−3^ M HCl (pH ~3) in a heating block (at 75 °C) for 48 h. The solution was then purified using 45–90 μm mesh filters to remove particulates (Elkay Laboratory Products, Basingstoke, UK) and 10 kDa MWCO filters (Pall Corporation, Port Washington, NY, USA) to remove low molecular weight contaminants^42^,^58^. The solution containing the >10 kDa fraction was frozen and lyophilized.

### Stable Isotope Analysis

Bone collagen stable isotope analyses were performed in duplicate on 0.5 mg collagen samples for *δ*^13^C and *δ*^15^N analyses and, where collagen yield allowed, 6.0 mg samples for *δ*^34^S. For scales, duplicate analyses were performed on collagen from two separate scales per individual. For *δ*^13^C and *δ*^15^N analyses, samples were combusted in tin capsules in an Elementar vario MICRO cube elemental analyzer coupled to an Isoprime isotope ratio mass spectrometer in continuous flow mode. Carbon and nitrogen isotopic compositions were calibrated relative to VPDB and AIR using USGS40 and USGS41. For *δ*^34^S analyses, samples were combusted in tin capsules with 1 mg of V_2_O_5_ in an Elementar vario MICRO cube elemental analyzer coupled to an Isoprime 100 isotope ratio mass spectrometer in continuous flow mode. Sulphur isotopic compositions were calibrated relative to VCDT using IAEA-S-1 and NBS-127.

### Sample Integrity

Sample integrity was assessed based on well-established criteria: collagen yields, C/N, C/S, and N/S ratios, and elemental percent values^43^,^44^,^45^. Samples from the Skyway and Robb sites produced collagen yields and C/N values suggesting poor collagen preservation and were therefore excluded. All other samples produced acceptable collagen integrity indicators, suggesting that stable isotope values have not been altered by diagenetic processes.

## Additional Information

**How to cite this article**: Guiry, E. J. *et al.* Lake Ontario salmon (*Salmo salar*) were not migratory: A long-standing historical debate solved through stable isotope analysis. *Sci. Rep.* **6**, 36249; doi: 10.1038/srep36249 (2016).

**Publisher’s note:** Springer Nature remains neutral with regard to jurisdictional claims in published maps and institutional affiliations.

## Supplementary Material

Supplementary Information
