# Supplementary material for: Lake Ontario salmon (Salmo salar) were not migratory: A long-standing historical debate solved through stable isotope analysis
Source: Sci Rep. 2016 Nov 8;6:36249. doi: 10.1038/srep36249 (PMC5099945; doi:10.1038/srep36249)
Supplement: Supplementary Information [file srep36249-s1.doc]

Supplementary Information

**Title:** Lake Ontario salmon (*Salmo salar*) were not migratory: A long-standing historical debate solved through stable isotope analysis

**Authors:** Eric J. Guiry, Suzanne Needs-Howarth, Kevin D. Friedland, Alicia L. Hawkins, Paul Szpak, Rebecca Macdonald, Michelle Courtemanche, Erling Holm, and Michael P. Richards

Overview of Historical Debate on Lake Ontario Salmon Migratory Behavior

Although comments and opinions from those who saw these animals in the flesh occasionally described firm beliefs that they were either potamodromous[^1-4^](#_ENREF_1) or anadromous[^5-8^](#_ENREF_5), most believed that it was possible that both forms were present.

Huntsman[^18^](#_ENREF_18) and later Webster[^19^](#_ENREF_19), for example, argued that Lake Ontario salmon must have been potamodromous, based mainly on the timing and seasonality of spawning as well as select records for their geographical distribution. Legendre et al. [^20^](#_ENREF_20) agree with their interpretations, citing a lack of historical references to salmon in the upper reaches of the St. Lawrence River (although a number of historical observations demonstrate the contrary)[^19^](#_ENREF_19) as well as the possibility that water temperatures in this section of the river were unsuitable for the species’ survival. Fox[^21^](#_ENREF_21) and Parson[^22^](#_ENREF_22), on the other hand, show a strong conviction that both types inhabited, and freely mixed in, Lake Ontario. Their argument is based on evidence that anadromous salmon were known to have migrated up a significant portion of the St. Lawrence River (at least to within a few tens of kilometers from the outlet of Lake Ontario) and that multiple races of Lake Ontario salmon existed (with distinguishing physical features and spawning seasons). Most recently, COSEWIC, using similar evidence for temporal variation in salmon spawning behavior, has come to the opposite conclusion, namely, that Lake Ontario salmon were probably only potamodromous. Still others, such as Dunfield[^25^](#_ENREF_25), who have reviewed the historical sources in detail, regard the controversy as an open question. The fact that the literature includes such opposing interpretations – even before the incorporation of the new information from archaeological datasets – shows the potential complexities in reconstructing the behaviors of extirpated or extinct fishes and underscores the need for new methods for assessing past animal life histories.

Scale Circuli Analyses

The spacings between scale circuli were measured from the end of the juvenile freshwater zone of the scale to the outer margin for three scales per specimen. These measurements provided a means of comparing the post-juvenile growth of historical specimens with the growth typical of ocean migrants[^26^](#_ENREF_26).

Sample Materials

Tables S1 and S2 provide contextual details for samples analyzed in this study.

Calibration, Accuracy, and Precision

Carbon and nitrogen isotopic and elemental compositions were determined using an Isoprime isotope ratio mass spectrometer (IRMS) coupled to an Elementar vario MICRO cube elemental analyzer (EA) at the Archaeology Isotope Laboratory (The University of British Columbia). Sample measurements were calibrated relative to VPDB (*δ*^13^C) and AIR (*δ*^15^N) (Table S4.1)[^27^](#_ENREF_27). Each CN analytical session included 8 or 9 measurements each of USGS40 and USGS41. Sulfur isotope and elemental compositions were determined using an Isoprime 100 IRMS coupled to an Elementar vario MICRO EA, also at the Archaeology Isotope Laboratory. *δ*^34^S values have been calibrated relative to VCDT (Table S3), using 2 or 3 measurements each of IAEA-S-1 and NBS-127 within each S analytical session.

The following internal standards were used to monitor accuracy and precision (Table S4). Each CN analytical session included 5 to 7 aliquots of at least two and more typically three check standards. The ^13^C and ^15^N isotope compositions reported here represent long-term averages calibrated to VPDB and AIR with USGS40 and USGS41: methionine (*n*=349), NIST 1577c (*n*=195), SUBC-1 (*n*=270), SRM-1 (*n*=132), and SRM-2 (*n*=119). Analytical sessions for S are typically shorter and included 1 to 3 aliquots of four check standards. The ^34^S compositions presented here represent long-term averages calibrated to VCDT with IAEA-S-1 and NBS-127: methionine (*n*=175), NIST 1577c (*n*=140), IAEA-S-3 (*n*=38), and casein protein (*n*=150).

Table S5 summarizes the mean and standard deviation of carbon and nitrogen isotopic compositions for all check standards, as well as the standard deviation for all calibration standards – the mean of the calibration standard for an individual run is predetermined to calibrate the data.

Table S6 summarizes the mean and standard deviation of sulfur isotope compositions for the check standards, as well as the standard deviation for all calibration standards – the mean of the calibration standard for an individual run is predetermined to calibrate the data.

All samples were analyzed for C and N in duplicate, with the exception of three samples for which there was not enough collagen (9530, 10258, 10686). The average difference between duplicate pairs was 0.07 ‰ for *δ*^13^C (range of <0.01 to 0.25) and 0.09 ‰ for *δ*^15^N (range of <0.01 to 0.46). Of the 25 samples analyzed for S, 16 provided enough collagen to be analyzed in duplicate. The average difference in *δ*^34^S values between duplicate pairs was 0.62 ‰ (range of 0.06 to 2.69).

Analytical Uncertainty

Analytical uncertainty for the *δ*^13^C and *δ*^15^N measurements of the samples (*u_s_*) was estimated using the following equation:


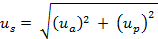
 **Equation S1**

Where *u_a_* represents accuracy or reproducibility and *u_p_* represents precision or repeatability. Accuracy (*u_a_*) is defined as the mean difference between the observed and known values for all of the check standards:


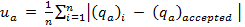
 **Equation S2**

The precision term (*u_p_*) is defined as the average standard deviation of all the check (*q_a_*) and calibration (*q_c_*) standards:


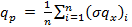
 **Equation S3**

In this case, because the mean difference between duplicate pairs is less than the average standard deviation of the check standards, no residual term was added to account for sample-specific heterogeneity.

Analytical uncertainty was determined to be ±0.11 ‰ for *δ*^13^C and ±0.12 ‰ for *δ*^15^N.

Analytical uncertainty was determined to be ±0.11 ‰ for *δ*^13^C, ±0.12 ‰ for *δ*^15^N, and 0.70 ‰ for *δ*^34^S values.

Tables

Table S1. Context information for archaeological salmon bones

| **SUBC** | **Site/Provenance** | **Region** | **Site Code** | **Element** | **Catalog No.** | **Time Period** |
| --- | --- | --- | --- | --- | --- | --- |
| 9350 | Joseph Picard | Lake Ontario | AlGs-376 | Dentary | 3412 | Pre-contact |
| 9351 | Joseph Picard | Lake Ontario | AlGs-376 | Dentary | 5531 | Pre-contact |
| 9352 | Joseph Picard | Lake Ontario | AlGs-376 | Dentary | 1725 | Pre-contact |
| 9353 | Joseph Picard | Lake Ontario | AlGs-376 | Dentary | 9145 | Pre-contact |
| 9354 | Joseph Picard | Lake Ontario | AlGs-376 | Dentary | 9500 | Pre-contact |
| 9354 | Joseph Picard | Lake Ontario | AlGs-376 | Dentary | 2634 | Pre-contact |
| 9355 | Trull | Lake Ontario | AlGq-67 | Dentary | 516 | Nineteenth C. |
| 9356 | Ashbridge | Lake Ontario | AjGt-1 | Maxilla | 2299 | Nineteenth C. |
| 9357 | Ashbridge | Lake Ontario | AjGt-1 | Caudal vertebra | 1789 | Nineteenth C. |
| 9359 | Ashbridge | Lake Ontario | AjGt-1 | Caudal vertebra | 436 | Nineteenth C. |
| 9361 | Ashbridge | Lake Ontario | AjGt-1 | Caudal vertebra | 2468 | Nineteenth C. |
| 9364 | Bishop's Block | Lake Ontario | AjGu-49 | Vertebra | 1668 | Nineteenth C. |
| 10254 | Mailhot-Curran | St. Lawrence | BgFn-2 | Vertebra | 304s | Pre-contact |
| 10255 | Mailhot-Curran | St. Lawrence | BgFn-2 | Vertebra | 307s | Pre-contact |
| 10256 | Mailhot-Curran | St. Lawrence | BgFn-2 | Vertebra | 843 | Pre-contact |
| 10257 | Mailhot-Curran | St. Lawrence | BgFn-2 | Ceratohyal | 1277s | Pre-contact |
| 10258 | Mailhot-Curran | St. Lawrence | BgFn-2 | Vertebra | 674s | Pre-contact |
| 10259 | Mailhot-Curran | St. Lawrence | BgFn-2 | Vertebra | 674s | Pre-contact |
| 10260 | Mailhot-Curran | St. Lawrence | BgFn-2 | Vertebra | 1441s | Pre-contact |
| 10261 | Mailhot-Curran | St. Lawrence | BgFn-2 | Vertebra | 62 | Pre-contact |
| 10262 | Mailhot-Curran | St. Lawrence | BgFn-2 | Vertebra | 639s | Pre-contact |
| 10642 | Yatsihsta | Lake Ontario | AlGs-452 | Pre-caudal vertebra | 821 | Pre-contact |
| 10643 | Yatsihsta | Lake Ontario | AlGs-452 | Pre-caudal vertebra | 986 | Pre-contact |
| 10649 | Yatsihsta | Lake Ontario | AlGs-452 | Pre-caudal vertebra | 1008 | Pre-contact |
| 10650 | Yatsihsta | Lake Ontario | AlGs-452 | Pre-caudal vertebra | 1178 | Pre-contact |
| 10651 | Yatsihsta | Lake Ontario | AlGs-452 | Pre-caudal vertebra | 1887 | Pre-contact |
| 10652 | Yatsihsta | Lake Ontario | AlGs-452 | Pre-caudal vertebra | 1924 | Pre-contact |
| 10653 | Yatsihsta | Lake Ontario | AlGs-452 | Pre-caudal vertebra | 2113 | Pre-contact |
| 10654 | Yatsihsta | Lake Ontario | AlGs-452 | Pre-caudal vertebra | 2856 | Pre-contact |
| 10655 | Yatsihsta | Lake Ontario | AlGs-452 | Vertebra | 3118 | Pre-contact |
| 10656 | Steven Patrick | Lake Ontario | BcGw-70 | Epihyal | 2009/3362 | Pre-contact |
| 10657 | Steven Patrick | Lake Ontario | BcGw-70 | Epihyal | 2010/3362 | Pre-contact |
| 10658 | Steven Patrick | Lake Ontario | BcGw-70 | Epihyal | 2011/3362 | Pre-contact |
| 10659 | Steven Patrick | Lake Ontario | BcGw-70 | Epihyal | 2013/3362 | Pre-contact |
| 10660 | Skyway | Lake Ontario | AhGw-278 | Vertebra | 40600 | Pre-contact |
| 10661 | Skyway | Lake Ontario | AhGw-278 | Pre-caudal vertebra | B460-464 | Pre-contact |
| 10664 | Robb | Lake Ontario | AlGt-4 | Vertebra | 1794 | Pre-contact |
| 10665 | Joseph Picard | Lake Ontario | AlGs-376 | Pre-caudal vertebra | 1912 | Pre-contact |
| 10666 | Joseph Picard | Lake Ontario | AlGs-376 | Atlas | 2176 | Pre-contact |
| 10667 | Joseph Picard | Lake Ontario | AlGs-376 | Vertebra | 2190 | Pre-contact |
| 10668 | Joseph Picard | Lake Ontario | AlGs-376 | Quadrate | 3065 | Pre-contact |
| 10669 | Joseph Picard | Lake Ontario | AlGs-376 | Ceratohyal | 5530 | Pre-contact |
| 10670 | Joseph Picard | Lake Ontario | AlGs-376 | Ceratohyal | 5548 | Pre-contact |
| 10671 | Joseph Picard | Lake Ontario | AlGs-376 | Dentary | 5607 | Pre-contact |
| 10672 | Joseph Picard | Lake Ontario | AlGs-376 | Vertebra | 5891 | Pre-contact |
| 10673 | Joseph Picard | Lake Ontario | AlGs-376 | Caudal vertebra | 5939 | Pre-contact |
| 10674 | Joseph Picard | Lake Ontario | AlGs-376 | Palatine | 6028 | Pre-contact |
| 10675 | Joseph Picard | Lake Ontario | AlGs-376 | Ceratohyal | 6054 | Pre-contact |
| 10676 | Joseph Picard | Lake Ontario | AlGs-376 | Caudal vertebra | 6176 | Pre-contact |
| 10677 | Joseph Picard | Lake Ontario | AlGs-376 | Vertebra | 6271 | Pre-contact |
| 10678 | Joseph Picard | Lake Ontario | AlGs-376 | Hyomandibular | 6466 | Pre-contact |
| 10679 | Joseph Picard | Lake Ontario | AlGs-376 | Caudal vertebra | 6661 | Pre-contact |
| 10680 | Joseph Picard | Lake Ontario | AlGs-376 | Pre-caudal vertebra | 7312 | Pre-contact |
| 10681 | Joseph Picard | Lake Ontario | AlGs-376 | Quadrate | 7497 | Pre-contact |
| 10682 | Joseph Picard | Lake Ontario | AlGs-376 | Quadrate | 7854 | Pre-contact |
| 10683 | Joseph Picard | Lake Ontario | AlGs-376 | Quadrate | 8379 | Pre-contact |
| 10684 | Joseph Picard | Lake Ontario | AlGs-376 | Vertebra | 8543 | Pre-contact |
| 10685 | Joseph Picard | Lake Ontario | AlGs-376 | Quadrate | 11395 | Pre-contact |
| 10687 | Joseph Picard | Lake Ontario | AlGs-376 | Vertebra | 12580 | Pre-contact |
| 10688 | Joseph Picard | Lake Ontario | AlGs-376 | Vertebra | 2224 | Pre-contact |
| 10689 | Joseph Picard | Lake Ontario | AlGs-376 | Vertebra | 2341 | Pre-contact |
| 10733 | Knowth | Ireland | E70:1975 | Vertebra | K75AB159 | Early Christian |
| 11703 | Summerstown | St. Lawrence | BgFp-1 | Vertebra | NA | Pre-contact |
| 11704 | Summerstown | St. Lawrence | BgFp-1 | Vertebra | NA | Pre-contact |
| 11705 | Summerstown | St. Lawrence | BgFp-1 | Vertebra | NA | Pre-contact |
| 11706 | Summerstown | St. Lawrence | BgFp-1 | Vertebra | NA | Pre-contact |
| 11707 | Summerstown | St. Lawrence | BgFp-1 | Vertebra | NA | Pre-contact |

Table S2. Context information for historical salmon mounts.

| **ROM Cat No.** | **SUBC No.** | **Royal Ontario Museum Specimen History** | **Behavioral Interpretation** | |
| --- | --- | --- | --- | --- |
|  |  |  | **Scale Pattern** | **δ^13^C Value** |
| 13184 | 10540 | This specimen is from Lake Ontario and was mounted in 1857. It was previously part of the Museum of the Department of Biology at the University of Toronto. Fork length = 690mm | Potamodromous | Potamodromous |
| 13185 | 10542 | This specimen is from Lake Ontario and was shown as part of an exhibition in the Museum of the Imperial Institute, London, England, until it was sent to the Royal Ontario Museum in 1929. It is thought to have been mounted at Wilmot's hatchery facilities at Newcastle, Ontario. Fork length = 785mm | Potamodromous | Potamodromous |
| 23235 | 10642 | This specimen is from Lake Ontario and was shown as part of an exhibition in the Museum of the Imperial Institute, London, England, until it was sent to the Royal Ontario Museum in 1929. It is thought to have been mounted at Wilmot's hatchery facilities at Newcastle, Ontario. A section of newspaper attached to the back of the specimen bears the publication date "Toronto, Sept. 4, 1882." Fork length = 652mm | Not analyzed | Potamodromous |
| 23236 | 10643 | This specimen is from Lake Ontario and was previously part of Wilmot's Museum at Newcastle, Ontario. Fork length = 760mm | Not analyzed | Potamodromous |
| 23237 | 10539 | This specimen is from Wilmot's Museum at Newcastle, Ontario, but was probably acquired in July, 1869, from the Moisie River in Quebec. Fork length = 892mm | Anadromous | Anadromous |
| 25390 | 10541 | This specimen was taken in Wilmot Creek near Lake Ontario in 1882 and was exhibited with other mounted Lake Ontario specimens in 1883 at the International Fisheries Exhibition in England. Fork length = 876mm | Potamodromous | Potamodromous |
| 101549 | 10538 | This specimen has lost it provenience information, but scale morphology patterns and stable isotope values are consistent with a St. Lawrence region origin. Fork length = 1100mm | Anadromous | Anadromous |

Table S3. Standard reference materials used for calibration of *δ*^13^C relative to VPDB, *δ*^15^N relative to AIR, and *δ*^34^S to VCDT.

| Standard | Material | Accepted *δ*^13^C  (‰, VPDB) | Accepted *δ*^15^N  (‰, AIR) | Accepted *δ*^34^S  (‰, VCDT) |
| --- | --- | --- | --- | --- |
| USGS40 | Glutamic Acid | −26.389 | −4.52 |  |
| USGS41  IAEA-S-1  NBS-127 | Glutamic Acid  Silver Sulfide  Barium Sulfate | +37.626 | +47.57 | −0.30  +20.3 |
|  |  |  |  |  |

Table S4. Standard reference materials used to monitor internal accuracy and precision.

| Standards | Material | Mean *δ*^13^C  (‰, VPDB) | Mean *δ*^15^N  (‰, AIR) | Mean *δ*^34^S  (‰, VCDT) |
| --- | --- | --- | --- | --- |
| MET  NIST 1577c | Methionine  Bovine liver | −28.60±0.08  −17.51±0.10 | −5.04±0.15  +8.15±0.15 | +9.1±0.6  +1.7±0.5 |
| SUBC-1 | Seal bone collagen | −13.67±0.11 | +17.39±0.14 |  |
| SRM-1  IAEA-S-3 | Caribou bone collagen  Silver Sulfide | −19.31±0.11 | +1.81±0.11 | −31.6±0.9 |
| Casein | Casein protein |  |  | +6.3±0.6 |

Table S5. Mean and standard deviation of all check and calibration standards for all analytical sessions containing data presented in this paper. Note that means for calibration standards are not presented, as they are pre-determined to be equal to the known value.

| **RUN ID** | **Standard** | ***n*** | ***δ*^13^C** |  |  | ***δ*^15^N** |  |  | **%C** |  |  | **%N** |  |  |
| --- | --- | --- | --- | --- | --- | --- | --- | --- | --- | --- | --- | --- | --- | --- |
| G15-13 | MET | 7 | -28.64 | ± | 0.04 | -5.04 | ± | 0.16 | 38.58 | ± | 0.42 | 8.95 | ± | 0.04 |
| G15-13 | NIST 1577c | 5 | -17.57 | ± | 0.03 | 8.08 | ± | 0.08 | 47.57 | ± | 0.89 | 9.76 | ± | 0.22 |
| G15-13 | SUBC-1 | 6 | -13.66 | ± | 0.08 | 17.37 | ± | 0.10 | 41.07 | ± | 1.62 | 15.04 | ± | 0.67 |
| G15-13 | USGS40 | 9 |  | ± | 0.06 |  | ± | 0.14 | 39.29 | ± | 0.38 | 9.14 | ± | 0.10 |
| G15-13 | USGS41 | 9 |  | ± | 0.22 |  | ± | 0.07 | 40.26 | ± | 0.37 | 9.36 | ± | 0.09 |
| G15-16 | MET | 7 | -28.64 | ± | 0.06 | -5.01 | ± | 0.14 | 38.97 | ± | 3.11 | 9.00 | ± | 0.65 |
| G15-16 | SUBC-1 | 6 | -13.61 | ± | 0.04 | 17.38 | ± | 0.08 | 40.11 | ± | 1.22 | 14.82 | ± | 0.42 |
| G15-16 | USGS40 | 9 |  | ± | 0.05 |  | ± | 0.11 | 37.94 | ± | 1.03 | 8.84 | ± | 0.21 |
| G15-16 | USGS41 | 9 |  | ± | 0.13 |  | ± | 0.14 | 39.49 | ± | 0.56 | 9.23 | ± | 0.12 |
| G15-19 | MET | 7 | -28.63 | ± | 0.03 | -5.16 | ± | 0.20 | 39.38 | ± | 1.92 | 9.04 | ± | 0.43 |
| G15-19 | NIST 1577c | 5 | -17.53 | ± | 0.02 | 8.13 | ± | 0.04 | 46.88 | ± | 0.35 | 9.58 | ± | 0.12 |
| G15-19 | SUBC-1 | 6 | -13.65 | ± | 0.09 | 17.35 | ± | 0.06 | 41.25 | ± | 1.39 | 15.01 | ± | 0.63 |
| G15-19 | USGS40 | 9 |  | ± | 0.06 |  | ± | 0.05 | 36.40 | ± | 4.77 | 8.38 | ± | 1.13 |
| G15-19 | USGS41 | 9 |  | ± | 0.17 |  | ± | 0.07 | 40.24 | ± | 0.18 | 9.29 | ± | 0.07 |
| G15-21 | MET | 7 | -28.62 | ± | 0.06 | -5.08 | ± | 0.22 | 38.67 | ± | 2.42 | 8.94 | ± | 0.51 |
| G15-21 | NIST 1577c | 5 | -17.52 | ± | 0.07 | 8.23 | ± | 0.09 | 46.57 | ± | 0.29 | 9.50 | ± | 0.09 |
| G15-21 | SUBC-1 | 6 | -13.67 | ± | 0.08 | 17.41 | ± | 0.10 | 42.05 | ± | 2.68 | 15.37 | ± | 1.02 |
| G15-21 | USGS40 | 9 |  | ± | 0.04 |  | ± | 0.06 | 38.77 | ± | 0.64 | 8.96 | ± | 0.19 |
| G15-21 | USGS41 | 9 |  | ± | 0.10 |  | ± | 0.13 | 40.11 | ± | 0.22 | 9.30 | ± | 0.08 |
| G15-22 | MET | 7 | -28.65 | ± | 0.04 | -5.02 | ± | 0.12 | 39.48 | ± | 2.73 | 8.95 | ± | 0.44 |
| G15-22 | NIST 1577c | 5 | -17.47 | ± | 0.18 | 8.16 | ± | 0.15 | 47.48 | ± | 0.92 | 9.68 | ± | 0.23 |
| G15-22 | SUBC-1 | 6 | -13.73 | ± | 0.08 | 17.37 | ± | 0.09 | 40.50 | ± | 1.68 | 14.81 | ± | 1.03 |
| G15-22 | USGS40 | 9 |  | ± | 0.11 |  | ± | 0.10 | 38.85 | ± | 0.16 | 8.74 | ± | 0.28 |
| G15-22 | USGS41 | 9 |  | ± | 0.31 |  | ± | 0.99 | 39.68 | ± | 0.37 | 8.97 | ± | 0.23 |
| G15-23 | MET | 7 | -28.64 | ± | 0.05 | -5.01 | ± | 0.11 | 40.11 | ± | 1.34 | 9.27 | ± | 0.29 |
| G15-23 | NIST 1577c | 2 | -17.52 | ± | 0.05 | 8.23 | ± | 0.06 | 48.39 | ± | 2.35 | 9.82 | ± | 0.36 |
| G15-23 | SRM-1 | 6 | -19.32 | ± | 0.03 | 1.87 | ± | 0.08 | 41.21 | ± | 0.56 | 14.70 | ± | 0.37 |
| G15-23 | SRM-2 | 5 | -14.69 | ± | 0.06 | 15.61 | ± | 0.12 | 42.05 | ± | 1.64 | 14.95 | ± | 0.55 |
| G15-23 | USGS40 | 8 |  | ± | 0.08 |  | ± | 0.10 | 39.42 | ± | 2.25 | 9.10 | ± | 0.60 |
| G15-23 | USGS41 | 8 |  | ± | 0.06 |  | ± | 0.11 | 41.14 | ± | 0.40 | 9.49 | ± | 0.06 |
| G15-29 | MET | 7 | -28.61 | ± | 0.04 | -5.03 | ± | 0.05 | 40.20 | ± | 1.87 | 9.36 | ± | 0.41 |
| G15-29 | NIST 1577c | 5 | -17.54 | ± | 0.01 | 8.13 | ± | 0.03 | 46.65 | ± | 5.72 | 9.60 | ± | 1.18 |
| G15-29 | SRM-1 | 6 | -19.32 | ± | 0.07 | 1.80 | ± | 0.06 | 43.03 | ± | 2.33 | 15.36 | ± | 0.89 |
| G15-29 | USGS40 | 9 |  | ± | 0.04 |  | ± | 0.06 | 37.29 | ± | 3.93 | 8.72 | ± | 0.91 |
| G15-29 | USGS41 | 9 |  | ± | 0.16 |  | ± | 0.18 | 40.41 | ± | 1.28 | 9.42 | ± | 0.30 |
| G16-01 | MET | 6 | -28.64 | ± | 0.21 | -5.03 | ± | 0.09 | 40.27 | ± | 3.98 | 9.42 | ± | 0.83 |
| G16-01 | NIST 1577c | 5 | -17.45 | ± | 0.08 | 7.92 | ± | 0.18 | 47.48 | ± | 0.97 | 9.89 | ± | 0.20 |
| G16-01 | SRM-1 | 6 | -19.35 | ± | 0.23 | 1.81 | ± | 0.03 | 43.23 | ± | 0.82 | 15.49 | ± | 0.37 |
| G16-01 | USGS40 | 9 |  | ± | 0.20 |  | ± | 0.11 | 37.28 | ± | 3.75 | 8.73 | ± | 0.91 |
| G16-01 | USGS41 | 9 |  | ± | 0.36 |  | ± | 0.12 | 40.38 | ± | 0.61 | 9.48 | ± | 0.14 |
| G16-02 | MET | 7 | -28.64 | ± | 0.20 | -5.03 | ± | 0.11 | 39.76 | ± | 3.30 | 9.10 | ± | 0.68 |
| G16-02 | NIST 1577c | 5 | -17.47 | ± | 0.07 | 8.13 | ± | 0.08 | 47.17 | ± | 0.69 | 9.59 | ± | 0.16 |
| G16-02 | SRM-1 | 6 | -19.40 | ± | 0.22 | 1.82 | ± | 0.05 | 41.71 | ± | 3.26 | 14.79 | ± | 1.27 |
| G16-02 | USGS40 | 9 |  | ± | 0.21 |  | ± | 0.13 | 36.92 | ± | 2.89 | 8.43 | ± | 0.66 |
| G16-02 | USGS41 | 9 |  | ± | 0.28 |  | ± | 0.12 | 40.34 | ± | 0.55 | 9.26 | ± | 0.18 |
| G16-04 | MET | 8 | -28.58 | ± | 0.04 | -5.08 | ± | 0.09 | 39.16 | ± | 2.69 | 9.10 | ± | 0.49 |
| G16-04 | NIST 1577c | 5 | -17.55 | ± | 0.04 | 8.14 | ± | 0.11 | 47.72 | ± | 0.82 | 9.80 | ± | 0.23 |
| G16-04 | SRM-1 | 6 | -19.34 | ± | 0.04 | 1.78 | ± | 0.05 | 43.20 | ± | 1.33 | 15.48 | ± | 0.45 |
| G16-04 | USGS40 | 8 |  | ± | 0.05 |  | ± | 0.06 | 35.82 | ± | 2.94 | 8.33 | ± | 0.65 |
| G16-04 | USGS41 | 9 |  | ± | 0.12 |  | ± | 0.20 | 39.07 | ± | 1.12 | 9.16 | ± | 0.27 |

Table S6. Mean and standard deviation of check and calibration standards for all sulfur analytical sessions containing data presented in this paper. Note that means for calibration standards are not presented, as they are pre-determined to be equal to the known value.

| **RUN ID** | **Standard** | ***n*** | ***δ*^34^S** |  |  | **%S** |  |  |
| --- | --- | --- | --- | --- | --- | --- | --- | --- |
| S-G16-01 | MET | 2 | 8.86 | ± | 0.48 | 20.09 | ± | 2.65 |
| S-G16-01 | NIST 1577b | 3 | 1.92 | ± | 0.29 | 0.76 | ± | 0.03 |
| S-G16-01 | Casein | 3 | 6.21 | ± | 1.10 | 0.71 | ± | 0.07 |
| S-G16-01 | IAEA-S-3 | 2 | -32.17 | ± | 1.86 | 13.85 | ± | 0.00 |
| S-G16-01 | NBS-127 | 3 |  | ± | 0.57 | 13.25 | ± | 0.62 |
| S-G16-01 | IAEA-S-1 | 2 |  | ± | 0.67 | 12.49 | ± | 0.53 |
| S-G16-02 | MET | 2 | 8.31 | ± | 0.09 | 21.99 | ± | 0.77 |
| S-G16-02 | NIST 1577b | 3 | 1.68 | ± | 0.89 | 0.76 | ± | 0.03 |
| S-G16-02 | Casein | 3 | 6.49 | ± | 0.81 | 0.79 | ± | 0.05 |
| S-G16-02 | IAEA-S-3 | 2 | -30.37 | ± | 2.09 | 13.35 | ± | 0.59 |
| S-G16-02 | NBS-127 | 2 |  | ± | 0.34 | 13.05 | ± | 0.36 |
| S-G16-02 | IAEA-S-1 | 2 |  | ± | 1.07 | 13.20 | ± | 0.97 |
| S-G16-03 | MET | 2 | 7.27 | ± | 0.01 | 16.38 | ± | 0.97 |
| S-G16-03 | Casein | 1 | 5.11 | ± |  | 0.52 | ± |  |
| S-G16-03 | IAEA-S-3 | 1 | -35.44 | ± |  | 16.81 | ± |  |
| S-G16-03 | NBS-127 | 2 |  | ± | 0.79 | 13.08 | ± | 3.93 |
| S-G16-03 | IAEA-S-1 | 1 |  |  |  | 7.89 |  |  |

Table S7. Collagen integrity and stable carbon and nitrogen isotope data for samples analyzed in this study.

| **Run ID** | **Sample (A)** | ***δ*^13^C** | ***δ*^15^N** | **%C** | **%N** | **C:N** | **Run ID** | **Sample (B)** | ***δ*^13^C** | ***δ*^15^N** | **%C** | **%N** | **C:N** | **|*Δ*| between Duplicate Pairs** | | | | |
| --- | --- | --- | --- | --- | --- | --- | --- | --- | --- | --- | --- | --- | --- | --- | --- | --- | --- | --- |
|  |  |  |  |  |  |  |  |  |  |  |  |  |  | ***δ*^13^C** | ***δ*^15^N** | **%C** | **%N** | **C:N** |
| G15-16 | 9350 | -19.89 | 10.06 | 38.19 | 13.47 | 3.31 |  |  |  |  |  |  |  |  |  |  |  |  |
| G15-13 | 9351 | -19.67 | 10.23 | 40.03 | 14.06 | 3.32 | G15-16 | 9351 b | -19.64 | 10.45 | 39.24 | 14.07 | 3.25 | 0.03 | 0.22 | 0.79 | 0.01 | 0.07 |
| G15-13 | 9352 | -19.73 | 10.44 | 39.70 | 14.07 | 3.29 | G15-16 | 9352 b | -19.73 | 10.26 | 39.10 | 13.88 | 3.29 | 0.00 | 0.18 | 0.60 | 0.19 | 0.00 |
| G15-13 | 9353 | -20.28 | 9.97 | 39.87 | 13.10 | 3.55 | G15-16 | 9353 b | -20.17 | 10.03 | 37.61 | 12.70 | 3.45 | 0.12 | 0.06 | 2.26 | 0.40 | 0.10 |
| G15-13 | 9354 | -20.65 | 9.59 | 38.31 | 12.65 | 3.53 | G15-16 | 9354 b | -20.66 | 9.73 | 36.97 | 12.33 | 3.50 | 0.01 | 0.14 | 1.33 | 0.32 | 0.03 |
| G15-21 | 9354 | -20.71 | 9.70 | 38.91 | 12.93 | 3.51 | G15-19 | 9354 b | -20.78 | 9.68 | 39.37 | 12.97 | 3.54 | 0.07 | 0.02 | 0.46 | 0.04 | 0.03 |
| G15-13 | 9355 | -20.10 | 10.42 | 33.16 | 11.22 | 3.45 | G15-16 | 9355 b | -20.12 | 10.53 | 38.75 | 13.06 | 3.46 | 0.02 | 0.11 | 5.58 | 1.84 | 0.01 |
| G15-13 | 9356 | -20.28 | 10.79 | 39.79 | 14.35 | 3.23 | G15-16 | 9356 b | -20.13 | 10.74 | 38.44 | 14.05 | 3.19 | 0.15 | 0.04 | 1.36 | 0.29 | 0.04 |
| G15-13 | 9357 | -20.35 | 11.01 | 39.50 | 12.82 | 3.59 | G15-16 | 9357 b | -20.25 | 10.86 | 38.00 | 12.57 | 3.53 | 0.09 | 0.16 | 1.50 | 0.25 | 0.07 |
| G15-13 | 9359 | -20.00 | 10.18 | 39.51 | 13.81 | 3.34 | G15-16 | 9359 b | -19.97 | 10.31 | 47.93 | 16.79 | 3.33 | 0.03 | 0.13 | 8.42 | 2.98 | 0.01 |
| G15-13 | 9361 | -20.49 | 10.31 | 37.43 | 13.14 | 3.32 | G15-16 | 9361 b | -20.51 | 10.43 | 35.95 | 12.88 | 3.25 | 0.02 | 0.11 | 1.48 | 0.26 | 0.07 |
| G15-13 | 9364 | -19.64 | 10.49 | 37.51 | 13.49 | 3.24 | G15-16 | 9364 b | -19.59 | 10.46 | 36.00 | 13.09 | 3.21 | 0.05 | 0.02 | 1.51 | 0.40 | 0.04 |
| G15-21 | 10254 | -22.12 | 10.03 | 40.58 | 14.65 | 3.23 | G15-19 | 10254 b | -22.00 | 10.06 | 40.26 | 14.36 | 3.27 | 0.12 | 0.03 | 0.32 | 0.29 | 0.04 |
| G15-21 | 10255 | -22.20 | 9.90 | 41.08 | 14.81 | 3.23 | G15-19 | 10255 b | -22.08 | 10.13 | 40.73 | 14.59 | 3.26 | 0.12 | 0.23 | 0.35 | 0.22 | 0.02 |
| G15-21 | 10256 | -22.68 | 10.28 | 39.70 | 13.25 | 3.49 | G15-19 | 10256 b | -22.85 | 10.37 | 38.87 | 12.74 | 3.56 | 0.16 | 0.09 | 0.83 | 0.51 | 0.06 |
| G15-21 | 10257 | -22.56 | 10.90 | 40.95 | 14.69 | 3.25 | G15-19 | 10257 b | -22.43 | 10.98 | 41.03 | 14.51 | 3.30 | 0.13 | 0.07 | 0.08 | 0.17 | 0.05 |
| G15-21 | 10258 | -15.11 | 13.72 | 38.98 | 13.28 | 3.42 |  |  |  |  |  |  |  |  |  |  |  |  |
| G15-21 | 10259 | -15.38 | 11.77 | 40.21 | 14.39 | 3.26 | G15-19 | 10259 b | -15.26 | 11.75 | 40.53 | 14.40 | 3.28 | 0.12 | 0.02 | 0.32 | 0.01 | 0.02 |
| G15-21 | 10260 | -15.16 | 10.53 | 40.54 | 14.27 | 3.31 | G15-19 | 10260 b | -15.10 | 10.67 | 40.32 | 14.10 | 3.34 | 0.06 | 0.14 | 0.22 | 0.17 | 0.02 |
| G15-21 | 10261 | -21.90 | 10.72 | 40.26 | 14.00 | 3.35 | G15-19 | 10261 b | -21.85 | 10.78 | 40.71 | 13.97 | 3.40 | 0.05 | 0.06 | 0.46 | 0.03 | 0.04 |
| G15-21 | 10262 | -22.10 | 10.33 | 39.34 | 13.70 | 3.35 | G15-19 | 10262 b | -21.92 | 10.43 | 39.99 | 13.90 | 3.35 | 0.18 | 0.10 | 0.65 | 0.21 | 0.00 |
| G15-29 | 10642 | -18.85 | 13.25 | 40.92 | 14.71 | 3.24 | G15-29 | 10642 b | -18.82 | 13.35 | 41.15 | 14.86 | 3.23 | 0.02 | 0.10 | 0.23 | 0.15 | 0.01 |
| G15-29 | 10643 | -19.91 | 13.07 | 41.28 | 14.78 | 3.26 | G15-29 | 10643 b | -19.86 | 13.16 | 41.13 | 14.70 | 3.26 | 0.05 | 0.09 | 0.16 | 0.08 | 0.01 |
| G16-01 | 10649 | -19.62 | 10.28 | 40.69 | 14.62 | 3.24 | G16-02 | 10649 b | -19.65 | 10.29 | 41.03 | 14.37 | 3.33 | 0.03 | 0.01 | 0.34 | 0.25 | 0.08 |
| G16-01 | 10650 | -19.61 | 10.30 | 41.71 | 14.80 | 3.29 | G16-02 | 10650 b | -19.59 | 10.23 | 41.93 | 14.87 | 3.29 | 0.02 | 0.07 | 0.22 | 0.07 | 0.00 |
| G16-01 | 10651 | -20.31 | 10.00 | 40.56 | 14.23 | 3.32 | G16-02 | 10651 b | -20.33 | 9.98 | 41.15 | 14.26 | 3.37 | 0.01 | 0.02 | 0.60 | 0.03 | 0.04 |
| G16-01 | 10652 | -20.73 | 10.46 | 41.07 | 13.39 | 3.58 | G16-02 | 10652 b | -20.73 | 10.42 | 40.93 | 13.22 | 3.61 | 0.00 | 0.04 | 0.14 | 0.16 | 0.03 |
| G16-01 | 10653 | -19.67 | 10.19 | 41.51 | 14.68 | 3.30 | G16-02 | 10653 b | -19.60 | 10.14 | 41.36 | 14.53 | 3.32 | 0.07 | 0.05 | 0.15 | 0.15 | 0.02 |
| G16-01 | 10654 | -20.10 | 10.12 | 42.17 | 14.30 | 3.44 | G16-02 | 10654 b | -20.26 | 10.11 | 41.66 | 14.11 | 3.44 | 0.16 | 0.02 | 0.51 | 0.18 | 0.00 |
| G16-01 | 10655 | -20.32 | 10.19 | 40.98 | 14.14 | 3.38 | G16-02 | 10655 b | -20.34 | 10.23 | 41.22 | 14.27 | 3.37 | 0.02 | 0.04 | 0.25 | 0.13 | 0.01 |
| G16-01 | 10656 | -20.34 | 10.20 | 42.85 | 14.36 | 3.48 | G16-02 | 10656 b | -20.31 | 10.16 | 42.03 | 13.96 | 3.51 | 0.03 | 0.03 | 0.81 | 0.40 | 0.03 |
| G16-01 | 10657 | -19.83 | 10.15 | 42.62 | 15.02 | 3.31 | G16-02 | 10657 b | -19.79 | 10.00 | 41.76 | 14.72 | 3.31 | 0.04 | 0.15 | 0.86 | 0.30 | 0.00 |
| G16-01 | 10658 | -19.44 | 12.90 | 42.26 | 14.36 | 3.43 | G16-02 | 10658 b | -19.52 | 12.84 | 41.39 | 13.89 | 3.48 | 0.08 | 0.07 | 0.88 | 0.47 | 0.04 |
| G16-01 | 10659 | -18.92 | 14.75 | 41.64 | 14.30 | 3.40 | G16-02 | 10659 b | -18.83 | 14.72 | 40.81 | 13.86 | 3.43 | 0.10 | 0.03 | 0.82 | 0.44 | 0.04 |
| G16-01 | 10660 | -19.42 | 12.91 | 41.74 | 14.08 | 3.46 | G16-02 | 10660 b | -19.40 | 12.92 | 41.07 | 13.80 | 3.47 | 0.03 | 0.01 | 0.67 | 0.27 | 0.01 |
| G16-01 | 10661 | -18.73 | 12.92 | 41.17 | 13.97 | 3.44 | G16-02 | 10661 b | -18.86 | 12.90 | 40.25 | 13.59 | 3.45 | 0.12 | 0.02 | 0.92 | 0.38 | 0.02 |
| G16-01 | 10664 | -20.02 | 10.53 | 41.32 | 14.52 | 3.32 | G16-02 | 10664 b | -20.02 | 10.52 | 41.12 | 14.31 | 3.35 | 0.00 | 0.01 | 0.20 | 0.21 | 0.03 |
| G16-01 | 10665 | -20.15 | 10.18 | 43.19 | 15.33 | 3.29 | G16-02 | 10665 b | -19.99 | 10.22 | 42.26 | 14.88 | 3.31 | 0.15 | 0.04 | 0.93 | 0.45 | 0.03 |
| G16-01 | 10666 | -20.22 | 9.74 | 42.90 | 15.03 | 3.33 | G16-02 | 10666 b | -20.26 | 9.77 | 41.80 | 14.62 | 3.33 | 0.05 | 0.04 | 1.10 | 0.41 | 0.00 |
| G16-01 | 10667 | -20.01 | 10.32 | 42.13 | 14.71 | 3.34 | G16-02 | 10667 b | -20.16 | 10.30 | 41.89 | 14.51 | 3.37 | 0.15 | 0.02 | 0.24 | 0.20 | 0.03 |
| G16-01 | 10668 | -19.96 | 10.39 | 41.91 | 14.87 | 3.29 | G16-02 | 10668 b | -19.98 | 10.30 | 41.30 | 14.48 | 3.33 | 0.02 | 0.09 | 0.61 | 0.39 | 0.04 |
| G16-01 | 10669 | -20.05 | 10.06 | 42.78 | 15.39 | 3.24 | G16-02 | 10669 b | -20.13 | 10.06 | 40.90 | 14.49 | 3.29 | 0.08 | 0.00 | 1.89 | 0.90 | 0.05 |
| G16-01 | 10670 | -20.13 | 10.72 | 43.55 | 15.39 | 3.30 | G16-02 | 10670 b | -20.01 | 10.65 | 41.88 | 14.55 | 3.36 | 0.12 | 0.07 | 1.67 | 0.84 | 0.06 |
| G16-01 | 10671 | -19.92 | 10.83 | 42.24 | 14.88 | 3.31 | G16-02 | 10671 b | -19.90 | 10.79 | 41.39 | 14.49 | 3.33 | 0.03 | 0.04 | 0.85 | 0.39 | 0.02 |
| G16-01 | 10672 | -20.84 | 9.79 | 42.16 | 14.57 | 3.37 | G16-02 | 10672 b | -20.87 | 9.78 | 41.54 | 14.04 | 3.45 | 0.03 | 0.01 | 0.62 | 0.53 | 0.08 |
| G16-01 | 10673 | -20.10 | 10.77 | 42.47 | 15.47 | 3.20 | G16-02 | 10673 b | -20.13 | 10.72 | 41.59 | 14.93 | 3.25 | 0.03 | 0.05 | 0.88 | 0.54 | 0.05 |
| G16-01 | 10674 | -19.63 | 10.76 | 41.64 | 15.08 | 3.22 | G16-02 | 10674 b | -19.69 | 10.71 | 41.32 | 14.63 | 3.29 | 0.06 | 0.06 | 0.32 | 0.45 | 0.07 |
| G16-01 | 10675 | -19.84 | 10.36 | 42.09 | 15.33 | 3.20 | G16-02 | 10675 b | -19.74 | 10.38 | 42.24 | 14.92 | 3.30 | 0.11 | 0.02 | 0.15 | 0.41 | 0.10 |
| G16-01 | 10676 | -19.77 | 10.10 | 41.76 | 14.88 | 3.27 | G16-02 | 10676 b | -19.76 | 9.98 | 41.83 | 14.71 | 3.32 | 0.01 | 0.13 | 0.07 | 0.17 | 0.04 |
| G16-01 | 10677 | -20.90 | 10.12 | 41.35 | 13.84 | 3.49 | G16-02 | 10677 b | -20.93 | 10.24 | 41.15 | 13.54 | 3.54 | 0.04 | 0.12 | 0.20 | 0.29 | 0.06 |
| G16-01 | 10678 | -19.63 | 10.23 | 43.04 | 15.38 | 3.26 | G16-02 | 10678 b | -19.64 | 10.12 | 41.61 | 14.99 | 3.24 | 0.01 | 0.12 | 1.43 | 0.39 | 0.03 |
| G16-01 | 10679 | -19.84 | 10.56 | 43.44 | 15.08 | 3.36 | G16-02 | 10679 b | -19.80 | 10.45 | 42.33 | 14.94 | 3.30 | 0.05 | 0.11 | 1.12 | 0.14 | 0.05 |
| G16-01 | 10680 | -19.36 | 10.55 | 43.04 | 15.23 | 3.30 | G16-02 | 10680 b | -19.55 | 10.50 | 41.55 | 14.77 | 3.28 | 0.19 | 0.05 | 1.49 | 0.46 | 0.02 |
| G16-01 | 10681 | -19.83 | 10.50 | 42.48 | 15.04 | 3.29 | G16-02 | 10681 b | -19.81 | 10.47 | 41.26 | 14.45 | 3.33 | 0.02 | 0.03 | 1.22 | 0.59 | 0.04 |
| G16-01 | 10682 | -19.71 | 10.02 | 41.65 | 14.71 | 3.30 | G16-02 | 10682 b | -19.65 | 9.97 | 40.35 | 13.99 | 3.36 | 0.06 | 0.05 | 1.30 | 0.72 | 0.06 |
| G16-01 | 10683 | -20.10 | 10.36 | 41.97 | 14.21 | 3.45 | G16-02 | 10683 b | -20.10 | 10.30 | 41.46 | 13.71 | 3.53 | 0.01 | 0.06 | 0.51 | 0.50 | 0.08 |
| G16-01 | 10684 | -19.95 | 10.08 | 42.58 | 14.58 | 3.40 | G16-02 | 10684 b | -19.88 | 10.04 | 41.91 | 14.14 | 3.46 | 0.07 | 0.03 | 0.67 | 0.44 | 0.05 |
| G16-01 | 10685 | -19.66 | 10.36 | 41.86 | 14.91 | 3.27 | G16-02 | 10685 b | -19.67 | 10.23 | 41.46 | 14.50 | 3.34 | 0.00 | 0.13 | 0.40 | 0.42 | 0.06 |
| G16-01 | 10687 | -19.55 | 10.23 | 42.41 | 15.42 | 3.21 | G16-02 | 10687 b | -19.55 | 9.92 | 41.99 | 15.02 | 3.26 | 0.01 | 0.32 | 0.42 | 0.39 | 0.05 |
| G16-01 | 10688 | -20.57 | 10.57 | 41.16 | 13.76 | 3.49 | G16-02 | 10688 b | -20.66 | 10.54 | 40.85 | 13.48 | 3.53 | 0.09 | 0.03 | 0.30 | 0.28 | 0.05 |
| G16-01 | 10689 | -20.13 | 10.05 | 41.47 | 14.48 | 3.34 | G16-02 | 10689 b | -20.18 | 9.67 | 41.78 | 14.22 | 3.43 | 0.05 | 0.37 | 0.31 | 0.27 | 0.09 |
| G16-04 | 10733 | -14.61 | 9.99 | 42.21 | 14.87 | 3.31 | G16-04 | 10733 b | -14.61 | 10.01 | 42.08 | 14.98 | 3.28 | 0.00 | 0.01 | 0.12 | 0.11 | 0.03 |
| G16-04 | 11703 | -15.26 | 9.94 | 40.70 | 14.37 | 3.30 | G16-04 | 11703 b | -15.31 | 10.01 | 41.09 | 14.56 | 3.29 | 0.05 | 0.06 | 0.39 | 0.18 | 0.01 |
| G16-04 | 11704 | -15.87 | 9.84 | 40.79 | 13.83 | 3.44 | G16-04 | 11704 b | -15.87 | 10.07 | 40.01 | 13.66 | 3.41 | 0.01 | 0.23 | 0.78 | 0.17 | 0.02 |
| G16-04 | 11705 | -16.63 | 9.96 | 40.08 | 12.70 | 3.68 | G16-04 | 11705 b | -16.62 | 10.01 | 39.75 | 12.81 | 3.62 | 0.01 | 0.05 | 0.33 | 0.12 | 0.06 |
| G16-04 | 11706 | -16.60 | 9.92 | 44.52 | 14.54 | 3.57 | G16-04 | 11706 b | -16.59 | 9.99 | 36.14 | 11.91 | 3.54 | 0.01 | 0.08 | 8.38 | 2.63 | 0.03 |
| G16-04 | 11707 | -15.96 | 9.81 | 39.64 | 13.66 | 3.38 | G16-04 | 11707 b | -15.88 | 9.83 | 39.26 | 13.65 | 3.35 | 0.07 | 0.02 | 0.38 | 0.01 | 0.03 |
| G15-22 | 10538-1 | -15.70 | 11.45 | 38.73 | 13.81 | 3.27 | G15-23 | 10538-1 b | -15.48 | 11.70 | 40.17 | 14.22 | 3.29 | 0.22 | 0.24 | 1.44 | 0.41 | 0.02 |
| G15-23 | 10538-2 | -15.53 | 11.47 | 40.73 | 14.67 | 3.24 | G15-23 | 10538-2 b | -15.67 | 11.55 | 40.74 | 14.60 | 3.25 | 0.13 | 0.08 | 0.00 | 0.07 | 0.02 |
| G15-22 | 10539-1 | -15.75 | 11.50 | 40.04 | 14.00 | 3.34 | G15-23 | 10539-1 b | -15.66 | 11.23 | 40.99 | 14.27 | 3.35 | 0.09 | 0.27 | 0.95 | 0.28 | 0.01 |
| G15-23 | 10539-2 | -15.72 | 11.52 | 40.89 | 14.33 | 3.33 | G15-23 | 10539-2 b | -15.48 | 11.06 | 40.96 | 14.35 | 3.33 | 0.25 | 0.46 | 0.07 | 0.02 | 0.00 |
| G15-22 | 10540-1 | -20.06 | 12.53 | 39.33 | 13.90 | 3.30 | G15-23 | 10540-1 b | -19.90 | 12.73 | 40.53 | 14.22 | 3.32 | 0.16 | 0.19 | 1.20 | 0.33 | 0.02 |
| G15-23 | 10540-2 | -19.93 | 12.55 | 41.20 | 14.43 | 3.33 | G15-23 | 10540-2 b | -19.81 | 12.19 | 41.16 | 14.52 | 3.31 | 0.12 | 0.36 | 0.04 | 0.09 | 0.02 |
| G15-22 | 10541-1 | -19.55 | 13.68 | 39.08 | 14.10 | 3.23 | G15-23 | 10541-1 b | -19.50 | 13.69 | 40.44 | 14.45 | 3.26 | 0.05 | 0.01 | 1.36 | 0.35 | 0.03 |
| G15-23 | 10541-2 | -19.98 | 12.47 | 40.92 | 14.53 | 3.28 | G15-23 | 10541-2 b | -19.98 | 12.63 | 40.86 | 14.43 | 3.30 | 0.01 | 0.16 | 0.06 | 0.10 | 0.02 |
| G15-22 | 10542-1 | -19.82 | 13.05 | 38.93 | 13.76 | 3.30 | G15-23 | 10542-1 b | -19.70 | 13.13 | 41.04 | 14.32 | 3.34 | 0.12 | 0.08 | 2.11 | 0.56 | 0.04 |
| G15-23 | 10542-2 | -19.70 | 12.54 | 40.91 | 14.31 | 3.33 | G15-23 | 10542-2 b | -19.81 | 12.51 | 41.00 | 14.28 | 3.35 | 0.11 | 0.03 | 0.08 | 0.04 | 0.02 |
| G15-29 | 10642 | -18.85 | 13.25 | 40.92 | 14.71 | 3.24 | G15-29 | 10642 b | -18.82 | 13.35 | 41.15 | 14.86 | 3.23 | 0.02 | 0.10 | 0.23 | 0.15 | 0.01 |
| G15-29 | 10643 | -19.91 | 13.07 | 41.28 | 14.78 | 3.26 | G15-29 | 10643 b | -19.86 | 13.16 | 41.13 | 14.70 | 3.26 | 0.05 | 0.09 | 0.16 | 0.08 | 0.01 |

Table S8. Elemental and stable sulfur isotope data for samples analyzed in this study.

| **Run ID** | **Sample (A)** | ***δ*^34^S** | **%S** | **Run ID** | **Sample (B)** | ***δ*^34^S** | **%S** | **|*Δ*| between Duplicate Pairs** | |
| --- | --- | --- | --- | --- | --- | --- | --- | --- | --- |
|  |  |  |  |  |  |  |  | ***δ*^34^S** | **%S** |
| S-G16-01 | 9350 a | 11.20 | 0.56 | S-G16-01 | 9350 b | 11.62 | 0.55 | 0.43 | 0.01 |
| S-G16-01 | 9351 a | 8.16 | 0.58 |  |  |  |  |  |  |
| S-G16-01 | 9352 a | 8.70 | 0.61 |  |  |  |  |  |  |
| S-G16-01 | 9353 a | 8.40 | 0.61 |  |  |  |  |  |  |
| S-G16-01 | 9354 a | 10.36 | 0.56 |  |  |  |  |  |  |
| S-G16-03 | 9356 a | 6.18 | 0.34 | S-G16-03 | 9356 b | 5.89 | 0.42 | 0.29 | 0.08 |
| S-G16-01 | 10254 a | 3.30 | 0.50 | S-G16-01 | 10254 b | 3.74 | 0.54 | 0.44 | 0.03 |
| S-G16-01 | 10257 a | 3.57 | 0.53 | S-G16-01 | 10257 b | 3.75 | 0.53 | 0.18 | 0.00 |
| S-G16-01 | 10259 a | 14.98 | 0.52 | S-G16-01 | 10259 b | 15.61 | 0.52 | 0.62 | 0.00 |
| S-G16-01 | 10260 a | 14.86 | 0.52 | S-G16-01 | 10260 b | 14.58 | 0.49 | 0.28 | 0.03 |
| S-G16-01 | 10538 a | 16.72 | 0.72 | S-G16-01 | 10538 b | 16.85 | 0.75 | 0.13 | 0.03 |
| S-G16-01 | 10539 a | 16.90 | 0.74 | S-G16-01 | 10539 b | 16.78 | 0.76 | 0.12 | 0.02 |
| S-G16-02 | 10540 a | 8.88 | 0.79 |  |  |  |  |  |  |
| S-G16-01 | 10541 a | 8.83 | 0.71 | S-G16-01 | 10541 b | 9.93 | 0.76 | 1.11 | 0.06 |
| S-G16-01 | 10542 a | 9.69 | 0.72 | S-G16-01 | 10542 b | 9.81 | 0.74 | 0.12 | 0.02 |
| S-G16-03 | 10649 a | 9.12 | 0.36 |  |  |  |  |  |  |
| S-G16-02 | 10650 a | 11.31 | 0.55 | S-G16-02 | 10650 b | 11.37 | 0.54 | 0.06 | 0.01 |
| S-G16-02 | 10653 a | 8.82 | 0.56 | S-G16-02 | 10653 b | 6.13 | 0.58 | 2.69 | 0.02 |
| S-G16-02 | 10657 a | 8.38 | 0.62 | S-G16-02 | 10657 b | 8.89 | 0.62 | 0.51 | 0.00 |
| S-G16-02 | 10658 a | 7.07 | 0.56 |  |  |  |  |  |  |
| S-G16-02 | 10664 a | 5.83 | 0.59 |  |  |  |  |  |  |
| S-G16-03 | 10673 a | 9.94 | 0.34 | S-G16-03 | 10673 b | 10.33 | 0.33 | 0.39 | 0.01 |
| S-G16-02 | 10687 a | 8.83 | 0.58 | S-G16-02 | 10687 b | 6.58 | 0.58 | 2.25 | 0.00 |
| S-G16-02 | 10733 a | 14.92 | 0.62 | S-G16-02 | 10733 b | 15.23 | 0.62 | 0.31 | 0.00 |
| S-G16-02 | 11703 a | 14.86 | 0.61 |  |  |  |  |  |  |

**References**

1 Clinton, D. *Letters on the Natural History and Internal Resources of the State of New York*. (E. Bliss & E. White, 1822).

2 Goode, G. B. *The Fisheries and Fishery Industries of the United States: Section 1. Natural History of Useful Aquatic Animals*. (US Government Printing Office, 1884).

3 Smith, H., M. Report on an investigation of the fisheries of Lake Ontario. *Bulletin of US Fish Commision* **10**, 195-202 (1892).

4 Simcoe, E. The Diary of Mrs. *John Graves Simcoe*, 187 ((1911)).

5 DeKay, J. E. in *Natural History of New York, Part 4* 241-242 (Geological Survey, 1842).

6 Talbot, E. A. *Five Years' Residence in the Canadas*. Vol. 1 (Longman, Hurst, Rees, Orme, Brown and Green, 1824).

7 Guillet, E. C. *Early Life in Upper Canada*. 267 (University of Toronto Press, 1963, 1963).

8 Wright, R. R. *Commissioners' Report*. (Warwick & sons, 1892).

9 Adamson, W. A. The decrease, resoration, and preservation of salmon in Canada. *Canadian Journal of Industry, Science, and Arts* **2**, 1-7 (1857).

10 Atkins, C. G. On the salmon of eastern North America, and its artificial culture. *United States Commision of Fish and Fisheries Report of the Commissioner for*, 227-335 (1872).

11 Nettle, R. *The salmon fisheries of the St. Lawrence and its tributaries*. (sn], 1857).

12 Wilmot, S. Vol. 5 *Annual Report of of the Department of Marine and Fisheries* 84-94 (I. B. Taylor, Ottawa, 1869).

13 Wilmot, S. *Annual Report of of the Department of Marine and Fisheries* 271-277 (I. B. Taylor Ottawa, 1871).

14 Wilmot, S. *Annual Report of of the Department of Marine and Fisheries* 96-104 (I. B. Taylor, Ottawa, 1873).

15 Wilmot, S. *Annual Report of the Commisioner of Fisheries* 341-367 (Maclean, Roger & Co., Ottawa, 1879).

16 Wilmot, S. *Annual Report of the Commisioner of Fisheries* 6-35 (Maclean, Roger & Co., Ottawa, 1880).

17 Wilmot, S. *Annual Report of of the Department of Marine and Fisheries* 3-47 (Maclean, Roger & Co., Ottawa, 1882).

18 Huntsman, A. G. Why did the Lake Ontario salmon disappear? *Transations of the Royal Society of Canada, Section 5, Series 3* **38**, 83-100 (1944).

19 Webster, D. A. Early history of the Atlantic salmon in New York [Salmo salar, fish]. *New York Fish and Game Journal* (1982).

20 Legendre, V., Mongeau, J.-R., Leclerc, J. & Briseboise, J. *Les salmonidés des eaux de la Plaine de Montréal*. (Montreal: Ministère du loisir, de la chasse et de la pêche, Service de l'amenagement et de l'exploitation de la faune, Region administrative de Montreal, 1980).

21 Fox, W. S. The literature of Salmo salar in Lake Ontario and tributary streams. *Transactions of the Royal Society of Canada, Section II, Series* **3**, 45-55 (1930).

22 Parsons, J. W. History of salmon in the Great Lakes, 1850-1970. (US Bureau of Sport Fisheries and Wildlife, 1973).

23 COSEWIC. *Committee on the Status of Endagered Animals in Canada* (Ottawa, 2011).

24 COSEWIC. *Committee on the Status of Endagered Animals in Canada* (Ottawa, 2006).

25 Dunfield, R. *The Atlantic salmon in the history of North America*. (Department of Fisheries and Oceans, 1985).

26 Friedland, K. D., Moore, D. & Hogan, F. Retrospective growth analysis of Atlantic salmon (Salmo salar) from the Miramichi River, Canada. *Canadian Journal of Fisheries and Aquatic Sciences* **66**, 1294-1308, doi:10.1139/F09-077 (2009).

27 Qi, H., Coplen, T. B., Geilmann, H., Brand, W. A. & Böhlke, J. Two new organic reference materials for δ13C and δ15N measurements and a new value for the δ13C of NBS 22 oil. *Rapid Communications in Mass Spectrometry* **17**, 2483-2487 (2003).
